# The impact of major depressive disorder on glycaemic control in type 2 diabetes: a longitudinal cohort study using UK Biobank primary care records

**DOI:** 10.1186/s12916-024-03425-9

**Published:** 2024-05-29

**Authors:** Alexandra C. Gillett, Saskia P. Hagenaars, Dale Handley, Francesco Casanova, Katherine G. Young, Harry Green, Cathryn M. Lewis, Jess Tyrrell

**Affiliations:** 1 Social, Genetic and Developmental Psychiatry Centre, Institute of Psychiatry, Psychology and Neuroscience, King’s College London, London, UK; 2 NIHR Maudsley Biomedical Research Centre, South London and Maudsley NHS Trust, London, UK; 3 Clinical and Biomedical Science, Institute of Health and Life Sciences, University of Exeter, Exeter, UK; 4 Department of Medical and Molecular Genetics, Faculty of Life Sciences and Medicine, King’s College London, London, UK

**Keywords:** Type 2 diabetes, Major depressive disorder, HbA1c, Longitudinal, Epidemiology

## Abstract

**Background:**

This study evaluates longitudinal associations between glycaemic control, measured by mean and within-patient variability of glycated haemaglobin (HbA1c) levels, and major depressive disorder (MDD) in individuals with type 2 diabetes (T2D), focusing on the timings of these diagnoses.

**Methods:**

In UK Biobank, T2D was defined using self-report and linked health outcome data, then validated using polygenic scores. Repeated HbA1c measurements (mmol/mol) over the 10 years following T2D diagnosis were outcomes in mixed effects models, with disease duration included using restricted cubic splines. Four MDD exposures were considered: MDD diagnosis prior to T2D diagnosis (pre-T2D MDD), time between pre-T2D MDD diagnosis and T2D, new MDD diagnosis during follow-up (post-T2D MDD) and time since post-T2D MDD diagnosis. Models with and without covariate adjustment were considered.

**Results:**

T2D diagnostic criteria were robustly associated with T2D polygenic scores. In 11,837 T2D cases (6.9 years median follow-up), pre-T2D MDD was associated with a 0.92 increase in HbA1c (95% CI: [0.00, 1.84]), but earlier pre-T2D MDD diagnosis correlated with lower HbA1c. These pre-T2D MDD effects became non-significant after covariate adjustment. Post-T2D MDD individuals demonstrated increasing HbA1c with years since MDD diagnosis ($\beta =0.51$, 95% CI: [0.17, 0.86]). Retrospectively, across study follow-up, within-patient variability in HbA1c was 1.16 (95% CI: 1.13–1.19) times higher in post-T2D MDD individuals.

**Conclusions:**

The timing of MDD diagnosis is important for understanding glycaemic control in T2D. Poorer control was observed in MDD diagnosed post-T2D, highlighting the importance of depression screening in T2D, and closer monitoring for individuals who develop MDD after T2D.

**Supplementary Information:**

The online version contains supplementary material available at 10.1186/s12916-024-03425-9.

## Background

Major depressive disorder (MDD) and type 2 diabetes (T2D) are substantial global health burdens, occurring together at twice the frequency expected by chance [1]. Adults with MDD have a 37% higher risk of developing T2D [2], and people with T2D face a 15% higher risk of developing MDD [3].

For individuals with T2D, comorbid depression is associated with elevated risk of diabetic complications [4] and all-cause mortality [5]. The underlying mechanisms between these disorders and outcomes remain poorly understood, but could include lifestyle factors, non-adherence to T2D treatment, use of antidepressant medication or genetic factors [1].

One potential link between MDD and adverse outcomes in T2D is glycaemic control, with glycated haemoglobin (HbA1c) representing a reliable measure of long-term glycemia. Elevated and more variable HbA1c levels are associated with increased risk of long-term diabetic complications, such as stroke and cardiovascular disease [6–8]. Prior studies have shown mixed support for the association between MDD and HbA1c [1, 9], but many are limited to a cross-sectional design. A meta-analysis that considered longitudinal effects of depressive symptoms on HbA1c found that depressive symptoms associated with higher HbA1c, and so poorer glycaemic control, across a mean follow-up of three years (*n* = 3683 across six studies) [9].

Furthermore, the relative timing of MDD and T2D diagnoses may also impact T2D clinical characteristics. A cross-sectional study showed that depression diagnosed after T2D is linked with poorer glycaemic control, and a higher prevalence of diabetic complications, compared to T2D patients with no or with pre-existing depression [10].

Understanding the impact of MDD on glycaemic control across time is crucial for delivering appropriate clinical care to individuals living with both MDD and T2D. Longitudinal studies to date have not explored the relationship between the relative diagnostic timings of these disorders and HbA1c trends over time. To address this gap, our study uses UK Biobank (UKB) primary care records to perform extensive longitudinal modelling of HbA1c in people with T2D in a retrospective, observational study, using information on MDD diagnosis. In the UK, HbA1c is measured by a general practitioner (GP) every 3–6 months in individuals with T2D [11]. Therefore, the linked primary care data available in UKB provides a unique opportunity to test the longitudinal relationship between HbA1c (mean levels and variability) and MDD over a 10-year period following T2D diagnosis. Our analysis incorporates four MDD exposures:

1. pre-T2D MDD diagnosis (ever diagnosed with MDD prior to T2D),
2. time between pre-T2D MDD diagnosis and T2D,
3. time-varying post-T2D MDD (newly diagnosed with MDD during follow-up period), and
4. time since post-T2D MDD diagnosis.

By considering these exposures, we aim to gain a deeper understanding of how MDD and its timing in relation to T2D affect glycaemic control.

## Methods

### Study population

The UKB is a health study of ~ 500,000 individuals recruited between 2006 and 2010 in the United Kingdom, aged 40–70 [12]. Linked primary care records are available for ~ 230,000 individuals (46%), encompassing clinical events, blood test results and prescriptions, providing longitudinal patient information [13].

#### T2D Classification and Validation

UKB participants with primary care records were classified into T2D cases and controls. T2D cases met specific T2D diagnostic criteria (detailed below), while controls did not. The T2D diagnostic criteria were validated using T2D polygenic scores (PGSs) in European ancestry participants meeting genetic quality control criteria [14, 15] (Additional file 1: Methods S1).

#### Type 2 diabetes (T2D) diagnostic criteria

T2D cases were identified based on the presence of any two of the following: 1) a primary care diagnosis code for T2D (Additional file 2: Table S1), 2) an ICD9/ICD10 diagnosis code for T2D (Additional file 2: Table S2), 3) any HbA1c measurement > 48 mmol/mol (6.5%), 4) any prescription for glucose lowering medication (Additional file 1: Methods S2 [16], Additional file 2: Table S3), and 5) a self-reported diagnosis for T2D with reported age at onset > 35 years. T2D diagnosis date was then the earliest occurrence among the identified criteria. T2D cases were excluded if they had a primary care code specific to type 1 diabetes, an insulin prescription within a year of T2D diagnosis, or a prescription for multiple diabetic medications at T2D diagnosis (Additional file 1: Methods S2-S3). Individuals prescribed one diabetic medication (monotherapy) at T2D diagnosis were not excluded.

#### Exclusion criteria for longitudinal analysis

**Fig. 1:**
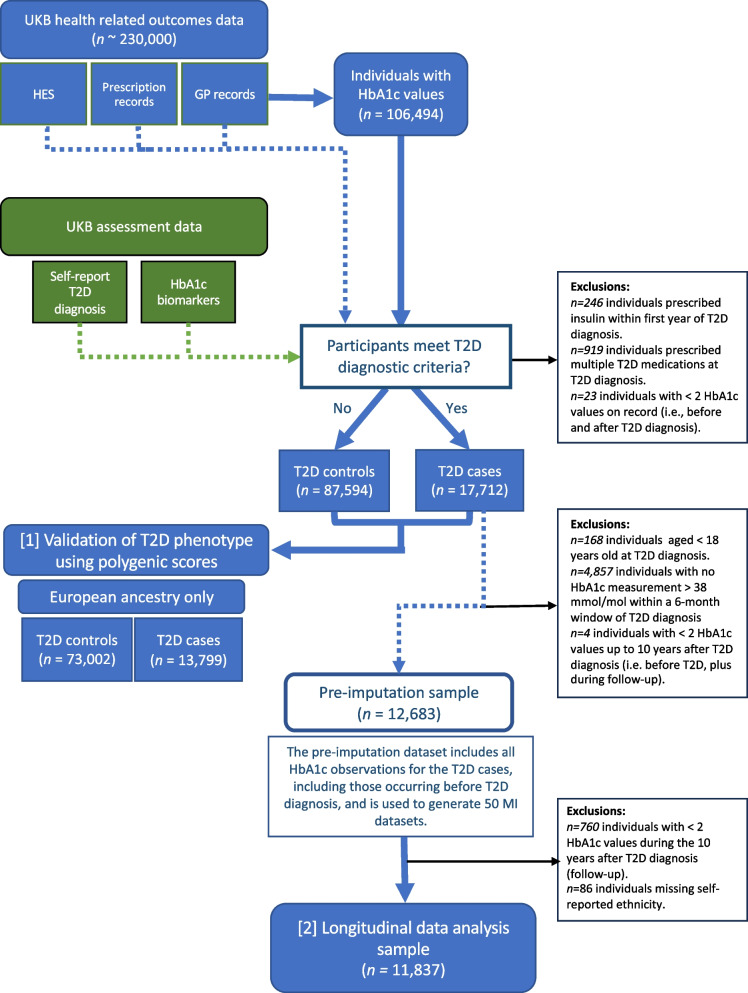
Flow diagram of UK Biobank (UKB) participant selection. T2D = type 2 diabetes. MDD = major depressive disorder. HES = hospital episode statistics. HbA1c = glycated haemaglobin. Validation of T2D diagnosis sample = T2D case and controls of European ancestry, meeting eligibility criteria outlined in Methods, including individual-level genetic analysis inclusion criteria (described in Additional File 1: Methods S1). Imputed datasets used for analyses (2) and (3) (adjusted mixed effects model for HbA1c over time and within-individual HbA1c variation)

For the longitudinal analysis, we excluded participants meeting any of the following criteria: 1) were a T2D control, 2) had fewer than two HbA1c measurements after T2D diagnosis, 3) had age at T2D diagnosis < 18 years, 4) had no recorded HbA1c measurements > 38 mmol/mol (3.5%) [17] within a six-month window of T2D diagnosis, 5) had diagnostic codes for bipolar, psychotic or substance-use disorders [18], 6) had a MDD diagnosis without a diagnosis date, or 7) were missing self-reported ethnicity (Fig. 1).

### Outcome measures

For the T2D diagnostic criteria validation, the outcome was T2D case–control status for UKB participants of European ancestry with primary care records available. For the longitudinal analysis, the outcome was repeated measures of HbA1c (mmol/mol) after T2D diagnosis. HbA1c data were taken from: 1) primary care records up to 2017 (Additional file 2: Table S4), where older observations recorded in %-units were converted to mmol/mol [19], and 2) all UKB biomarker assessments (2006–2016), where a validated correction was applied to account for lower average HbA1c values from the UKB biomarker panel compared to primary care [20] (Additional file 1: Methods S4). The indexing date was T2D diagnosis, with a maximum follow-up period of 10 years.

### Exposures

In the longitudinal analysis, four MDD exposure variables were considered simultaneously. Two were related to individuals diagnosed with MDD prior to T2D (pre-T2D MDD), and two were related to individuals diagnosed with MDD after their T2D diagnosis (post-T2D MDD).

*Pre-T2D MDD exposures:* (1) History of MDD at T2D diagnosis (MDD_index). This binary variable indicates whether an individual had ever received a MDD diagnosis at the index date. (2) Pre-T2D MDD duration at index (years). This semi-continuous variable quantifies the time between MDD diagnosis and T2D diagnosis when MDD_index equals 1, and is 0 otherwise. These time-invariant exposures were used to examine the impact of pre-T2D MDD on HbA1c, including interactions with T2D disease duration.

*Post-T2D MDD exposures:* (1) Change in MDD diagnosis (MDD_change). A time-varying binary variable indicating, at each observation time (*t*), whether an individual has been diagnosed with MDD between *t* and the index date. It captures information about individuals diagnosed with MDD during follow-up. For individuals with no MDD diagnosis occurring before *t*, and those diagnosed prior to T2D diagnosis, this variable is set to 0. (2) Post-T2D MDD duration (years). A time-varying, semi-continuous variable equalling the time between MDD diagnosis and *t* if MDD_change equals 1, and 0 otherwise. This variable allows post-T2D MDD participants to have different HbA1c time-slopes after their MDD diagnosis. Note, UKB participants classified as having a MDD diagnosis required at least two diagnostic codes for a depressive disorder or episode diagnosis in the linked primary care records [18] (Additional file 1: Methods S5).

### Covariates

Covariates were extracted from UKB assessments and/or primary care data. Covariates extracted from UKB initial assessments were: sex, assessment centre, self-reported ethnicity, Townend Deprivation Index (TDI), qualifications, ever smoked, and never consumed alcohol. Covariates extracted from both UKB assessments and primary care data were: age at T2D diagnosis, HbA1c at T2D diagnosis, body mass index (BMI) at T2D diagnosis (Additional file 1: Methods S6 [21, 22]), systolic (SBP) and diastolic (DBP) blood pressure at T2D diagnosis (Additional file 1: Methods S6), number of HbA1c, BMI and blood pressure measurements taken prior to T2D diagnosis, and T2D disease duration (time since diagnosis). Covariates extracted from primary care only were diabetic medications. Glucose lowering medication at each HbA1c observation was identified using prescription records up to three months prior to HbA1c measurement (Additional file 1: Methods S2). This information was grouped into four medication categories: 1) M0 (‘no medication’), 2) M1 (‘metformin or a single medication’/ monotherapy), 3) M2 (‘two medications’/ dual-therapy), and 4) M3 (either ‘3 or more medications’ or ‘insulin’). Two medication variables were then created. Firstly, a binary variable indicating monotherapy at T2D diagnosis versus no prescribed T2D medication (M0 vs M1 at diagnosis; recall exclusion criteria removed individuals prescribed multiple medications at diagnosis). Secondly, a time-varying medication variable using the four categories M0–M3.

Apart from T2D disease duration and time-varying medication, covariates were treated as baseline measurements, but it is important to note that the index date and UKB assessment dates are different. For example, measurements from UKB initial assessments (TDI, qualifications, etc.) were collected between 2006 and 2010, and 56% of individuals were diagnosed outside of this timeframe. However, results from models with and without UKB initial assessment covariates yielded similar conclusions (Additional file 2: Table S5). Further details on covariates are available in Additional file 2: Table S6 [18–23].

### Statistical analysis

All analyses were performed using R version 4.2.2 and visualised using ggplot2.

#### Validation of T2D diagnostic criteria

To validate the T2D definition, we tested whether PGSs for T2D [24] predicted T2D case–control status. PGSs, calculated using PRSice v2 [25, 26] at eleven *P*-value thresholds, were tested for association with T2D case–control status, adjusting for six genetic ancestry principal components, assessment centre and genetic batch effect (Additional file 1: Methods S7).

#### Longitudinal analysis

We employed linear mixed effects models (MEMs) to investigate longitudinal associations between HbA1c and MDD using the nlme package [27, 28]. All models incorporated random intercepts and time slopes, with a continuous-time autoregressive 1 (CAR1) residual correlation structure to account for autocorrelation. A series of three analyses were performed.

##### (1) Unadjusted model with selection

This analysis focused on the primary fixed effects of time (T2D disease duration) and the MDD exposures. Time was included using a restricted cubic spline (RCS) with four knots to allow for a non-linear temporal trend in mean HbA1c [29, 30]. Interactions between the pre-T2D MDD exposures and time-splines were considered, with the impact of post-T2D MDD on HbA1c time-slopes captured by post-T2D MDD duration. Selection of the final model was determined by Akaike Information Criterion (AIC) and likelihood ratio tests (LRTs). Models with semi-continuous exposures required their corresponding binary indicator to be included.

##### **(2) Adjusted model**

The selected unadjusted model was extended to include covariates, plus interactions between the time-splines and HbA1c at T2D diagnosis, BMI at T2D diagnosis and the medication variables.

##### **(3) Residual within-subject variation in HbA1c**

To explore the association between within-subject variation and MDD, the adjusted model was updated to allow residual variation to differ by MDD diagnosis variables. Firstly, we assessed if pre-T2D MDD (MDD_index) individuals had different residual variation compared to other participants. Secondly, we used MDD_change to investigate if post-T2D MDD individuals had different residual variation after their MDD diagnosis. Thirdly, a retrospective, time-invariant binary indicator for post-T2D MDD individuals was introduced to assess any differences in within-subject variation over all of follow-up. Please see Additional file 1: Methods S8 [27–30] for a mathematical explanation of these models. Likelihood ratio tests (LRTs) were used to test for MDD diagnosis-related heterogeneity in residual variation.

HbA1c at T2D diagnosis had a high level of missingness (40%), but all included participants had a HbA1c measurement within a six-month window of this date. Multiple imputation (MI) with the Amelia package [31] generated 50 MI datasets (Additional file 1: Methods S9) that were used in analyses (2) and (3). MEM estimates were pooled using Rubin’s method, with pooled Wald-like *P*-values presented for the fixed effects [32, 33]. Parameter estimates for RCSs are difficult to interpret. Therefore, in addition to a summary of the MDD exposure fixed effects (including *P*-values from t-tests), results are presented using plots of predicted HbA1c [34, 35]. Details of covariates included in MI and/ or MEMs are given in Additional file 2: Tables S6-S7.

## Results

### Validation of T2D diagnostic criteria

**Fig. 2:**
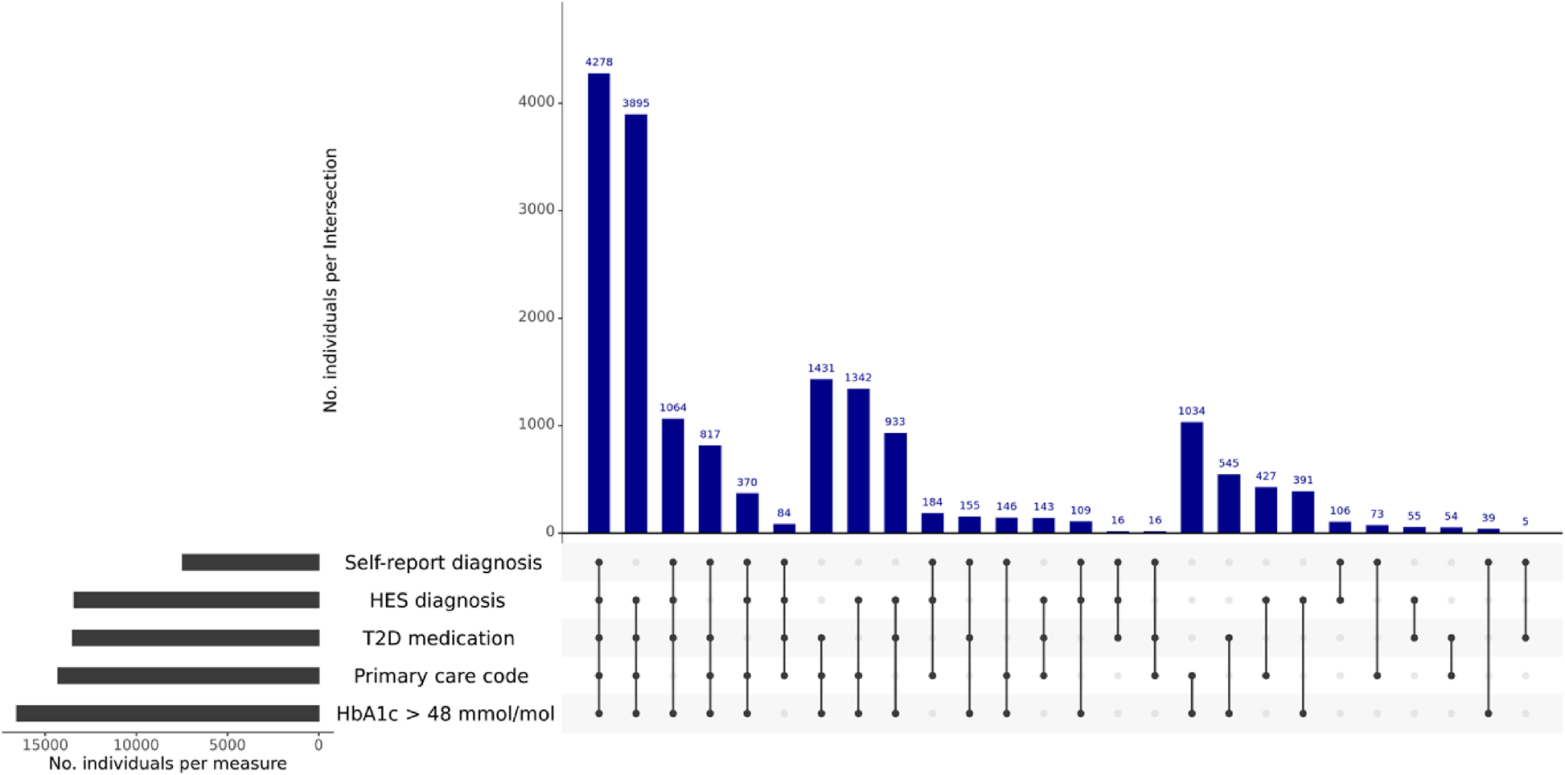
Contributions of each input to the T2D phenotype definition. Horizontal bars indicate the number of individuals who met criteria for the corresponding T2D sub-phenotypes. Vertical bars indicate the number of individuals endorsing combinations of the five T2D sub-phenotypes

The eligible UKB sample consisted of 17,712 individuals with T2D (Fig. 2), with 24% of individuals having evidence of T2D from all five data sources considered. Participants with T2D tended to be male (61%) with an average age at T2D diagnosis of 57 years (IQR: 51–64 years). Applying genetic quality control criteria to participants of European ancestry provided 13,799 T2D cases and 73,002 controls. T2D PGSs were significantly associated with T2D case–control status at all *P*-value thresholds considered and explained up to 2.8% of T2D liability (Additional file 3: Table S1).

### Longitudinal analysis

In total, 11,837 T2D cases were included, with a median follow-up time of 6.9 years (IQR: 3.5–9.3 years). Table 1 presents sample characteristics stratified by MDD subgroup: ‘no MDD’ (89% without an MDD diagnosis), ‘pre-T2D MDD’ (9% with MDD diagnosed prior to T2D) and ‘post-T2D MDD’ (2% diagnosed with MDD during the 10-year follow-up period). Groups with a diagnosis of MDD include a higher proportion of females and have higher median TDI, which is particularly notable in the post-T2D MDD group (Additional File 4: Figure S1). Additionally, the post-T2D MDD group had an earlier median age at T2D diagnosis, leading to a longer median follow-up time (Additional File 4: Figures S2-S3). This group also had the highest proportion of patients in medication group M3 (insulin and/or three or more T2D medications) by the end of follow-up. A higher proportion of the post-T2D MDD group are missing HbA1c at diagnosis (52% compared to 43% for the pre-T2D MDD group and 42% for the no MDD group). See Additional file 4: Figures S1-S2 and S4-S8 for density plots of continuous covariates included in the MEMs.

**Table 1:** Characteristics of the longitudinal analysis study participants, stratified by MDD subgroups. Columns present *N* (%) for categorical variables and median (IQR) for continuous variables

	**Total** **(*N* = 11,837)**	**No MDD** **(*N* = 10,492, 89%)**	**Pre-T2D MDD** **(*N* = 1119, 9%)**	**Post-T2D MDD** **(*N* = 226, 2%)**
**Female**	4775 (40.34)	4038 (38.49)	634 (56.66)	103 (45.58)
**Year of birth**	1948 (1943, 1954)	1947 (1943, 1953)	1949 (1945, 1955)	1950 (1945, 1956)
**Genetic ancestry**				
European	9953 (84.08)	8797 (83.84)	978 (87.4)	178 (78.76)
African	229 (1.93)	209 (1.99)	14 (1.25)	6 (2.65)
Admixed African American	54 (0.46)	48 (0.46)	4 (0.36)	2 (0.88)
East Asian	76 (0.64)	73 (0.70)	3 (0.27)	0 (0.00)
South Asian	700 (5.91)	638 (6.08)	43 (3.84)	19 (8.41)
Missing	825 (6.97)	727 (6.93)	77 (6.88)	21 (9.29)
**Measurements at T2D diagnosis (year of T2D diagnosis range: 1990 – 2017)**				
**Year**	2009 (2006, 2012)	2009 (2006, 2012)	2009 (2007, 2013)	2005 (2003, 2008)
**Age (years)**	60.4 (54.4, 65.4)	60.6 (54.6, 65.5)	59.4 (54.2, 64.6)	55.6 (49.2, 60.1)
**HbA1c (mmol/mm)**	53 (50, 62.41)	53 (50, 63)	52 (50, 59)	52.91 (50, 67.26)
Missing	4952 (41.83)	4353 (41.49)	482 (43.07)	117 (51.77)
No measurement within ± 2 weeks	3326 (28.1)	2920 (27.83)	323 (28.87)	83 (36.73)
**Prescribed monotherapy**	408 (3.45)	355 (3.38)	44 (3.93)	9 (3.98)
**BMI (kg/m^2)**	31.2 (27.96, 35.2)	31 (27.83, 35)	32.7 (28.93, 37.25)	31.42 (28.25, 36.7)
Missing	595 (5.03)	541 (5.16)	43 (3.84)	11 (4.87)
**Systolic blood pressure**	139 (130, 149)	139 (130, 150)	138 (128, 148)	138 (130, 149)
Missing	511 (4.32)	478 (4.56)	26 (2.32)	7 (3.1)
**Diastolic blood pressure**	81 (76, 89.5)	81 (76, 89)	81.5 (76, 90)	82 (76.75, 90)
Missing	511 (4.32)	478 (4.56)	26 (2.32)	7 (3.1)
**Prior no. of observations**	14 (6, 28)	13 (5, 27)	20 (11, 35)	10 (4, 22)
**Measurements at UK Biobank initial assessment (year of initial assessment range: 2006 – 2010)**				
** Self-reported ethnicity**				
White	10,596 (89.52)	9364 (89.25)	1042 (93.12)	190 (84.07)
Black	251 (2.12)	226 (2.15)	19 (1.7)	6 (2.65)
Asian	707 (5.97)	647 (6.17)	37 (3.31)	23 (10.18)
Chinese	40 (0.34)	37 (0.35)	3 (0.27)	0 (0)
Mixed race	77 (0.65)	65 (0.62)	10 (0.89)	2 (0.88)
Other	166 (1.4)	153 (1.46)	8 (0.71)	5 (2.21)
**TDI**	-1.35 (-3.19, 1.78)	-1.38 (-3.21, 1.76)	-1.28 (-3.15, 1.78)	-0.08 (-2.35, 3.3)
Missing	32 (0.27)	29 (0.28)	3 (0.27)	0 (0)
**Qualifications**				
College or University	2459 (20.77)	2205 (21.02)	222 (19.84)	32 (14.16)
Alevels/ASlevels/equivalent	1036 (8.75)	898 (8.56)	112 (10.01)	26 (11.5)
O levels/GCSEs/equivalent	2352 (19.87)	2089 (19.91)	216 (19.3)	47 (20.8)
CSEs/equivalent	581 (4.91)	503 (4.79)	64 (5.72)	14 (6.19)
NVQ/HND/HNC/equivalent	1066 (9.01)	942 (8.98)	102 (9.12)	22 (9.73)
Other	680 (5.74)	612 (5.83)	61 (5.45)	7 (3.1)
None of the above	3436 (29.03)	3044 (29.01)	318 (28.42)	74 (32.74)
Missing	227 (1.92)	199 (1.9)	24 (2.14)	4 (1.77)
**Ever smoked**	7505 (63.4)	6637 (63.26)	722 (64.52)	146 (64.6)
Missing	66 (0.56)	59 (0.56)	6 (0.54)	1 (0.44)
**Never consumed alcohol**	963 (8.14)	865 (8.24)	80 (7.15)	18 (7.96)
Missing	28 (0.24)	24 (0.23)	2 (0.18)	2 (0.88)
**MDD diagnosis**				
**Age (years)**			47.15 (39.59, 54.29)	58.32 (53.35, 63.11)
**Absolute time between MDD and T2D diagnosis (years)**			10.69 (4.89, 17.63)	3.24 (1.32, 5.18)
**Follow-up variables**				
**Follow-up time (years)**	6.89 (3.54, 9.31)	6.89 (3.54, 9.3)	6.33 (3.17, 8.98)	9.29 (7.16, 9.74)
**No. of HbA1c observations during follow-up**	12 (6, 18)	11 (6, 18)	11 (6, 18)	17.5 (13, 23)
**Ever been prescribed the following medications from diagnosis to end of follow-up**				
M1 (monotherapy)	8286 (70)	7317 (69.74)	791 (70.69)	178 (78.76)
M2 (dual therapy)	3894 (32.9)	3403 (32.43)	375 (33.51)	116 (51.33)
M3	1678 (14.18)	1451 (13.83)	161 (14.39)	66 (29.2)
Insulin	682 (5.76)	582 (5.55)	66 (5.9)	34 (15.04)

Genetic ancestry: defined as one of the five 1000 Genome super populations, genetically inferred using the ukbkings R package [23]. Prior no. of observations: total number of HbA1c, BMI and BP records observed prior to T2D diagnosis. UK Biobank initial assessment is not typically the same as T2D diagnosis date. Follow-up: Last available HbA1c measurement observed during the first 10 years since T2D diagnosis (up to 2017). Density plots for continuous variables used as covariates, and follow-up time, are available in Additional File 4: S1–S8

*IQR *interquartile range

#### Unadjusted model

**Fig. 3:**
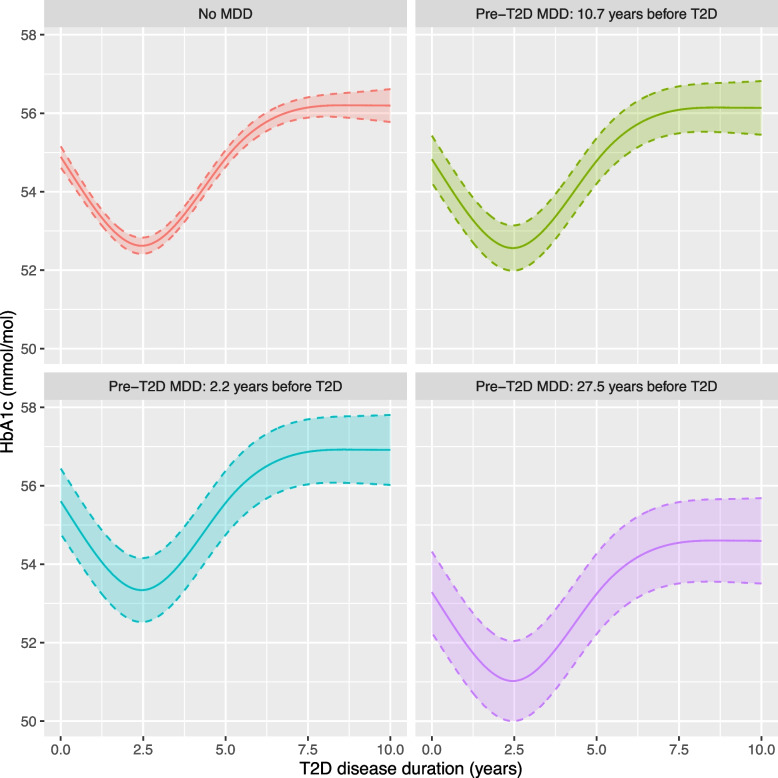
Predicted HbA1c (mmol/mol) with 95% confidence intervals from the unadjusted model, for four example individuals: no MDD and three pre-T2D MDD individuals diagnosed with MDD 2.2 (10th percentile), 10.7 (median) and 27.5 (90th percentile) years before T2D

The selected unadjusted model included time-splines and the four MDD exposures, with no significant interaction between the pre-T2D MDD exposures and time (*p* = 0.3072; Table 2). This implies the difference in mean HbA1c between pre-T2D MDD individuals and individuals without MDD depends on the time between MDD and T2D diagnoses, the effect of which is constant over T2D disease duration. Having a history of MDD prior to T2D is associated with a 0.92 mmol/mol increase (95% CI: [0.00, 1.84]) in HbA1c, but earlier onset of pre-T2D MDD is associated with lower HbA1c (Table 3). To illustrate this, Fig. 3 plots predicted HbA1c (mmol/mol) over time for four example individuals with either no MDD or with MDD diagnosed 2.2 years, 10.7 years, or 27.5 years before T2D (10th, 50th and 90th percentile of pre-T2D MDD duration at index respectively). Differences in HbA1c between these three pre-T2D MDD individuals and the no MDD individual at any point during follow-up are 0.72 (95% CI: [-0.11, 1.55]), -0.06 (95% CI: [-0.65, 0.54]) and -1.60 (95 CI: [-2.63, -0.57]) for the 10th, 50th and 90th percentile of pre-T2D MDD duration respectively. Those diagnosed with MDD ~ 10 years before T2D have HbA1c levels over time equivalent to those without MDD, with those diagnosed earlier having lower HbA1c and those diagnosed closer to their T2D diagnosis having higher HbA1c. Our example shows that only individuals diagnosed with MDD many decades before T2D have a 95% CI for difference in HbA1c relative to no MDD that excludes 0 (Additional file 3: Table S2a).

**Table 2:** Unadjusted model selection summary. Model 2, with mean HbA1c being a function of time (four knot restricted cubic spline for T2D disease duration), the pre-T2D MDD exposures and the post-T2D exposures, has the lowest AIC and is selected using likelihood ratio tests (LRTs)

Model	Included fixed effects							df	AIC	LRT	
	Time (splines)	Pre-T2D MDD exposures				Post-T2D MDD exposures				Models compared	*P*-value
		MDD_index		Duration		MDD_change	Duration				
		Main	Int	Main	Int						
1	✓	✓	✓	✓	✓	✓	✓	19	247,790.8		
**2**	✓	✓		✓		✓	✓	**13**	**247,786.0**	**2** vs 1	0.3072
3	✓	✓				✓	✓	11	247,794.0	3 vs **2**	0.0015
4	✓	✓		✓		✓		12	247,793.5	4 vs **2**	0.0020

*LRT* likelihood ratio test, performed using anova with mixed effects models estimated with maximum likelihood, *df* degrees of freedom (random effects and covariance parameters included in count). *AIC* Akaike Information Criterion (smaller is preferred). *Int* interaction between exposure and the time splines (4 knots). Pre-T2D MDD exposures: Duration is time between MDD and T2D diagnoses for individual with MDD_index equal to 1, and 0 otherwise. Post-T2D MDD exposures: Duration is time between HbA1c observation and MDD diagnosis when MDD_change equals 1, and 0 otherwise. See “Exposures” section for details. Bold number in ‘Models compared’ column is the model preferred by the LRT. Stop at model 4 because pre-T2D MDD and post-T2D MDD duration variables are selected for inclusion

**Table 3:** Fixed effect estimates for MDD exposure variables from the unadjusted and pooled adjusted linear mixed effects models

MDD Exposure Variables	Unadjusted model			Pooled adjusted model		
	**Beta**	**95% CI**	***P*-value**	**Beta**	**95% CI**	***P*-value**
**Pre-T2D MDD exposure variables**						
Pre-T2D MDD	0.92	( 0.00, 1.84)	0.0496	0.84	(-0.06, 1.73)	0.0678
Pre-T2D MDD duration to index (years)	-0.09	(-0.15, -0.04)	0.0015	-0.04	(-0.09, 0.02)	0.1637
**Post-T2D MDD exposure variables**						
Post-T2D MDD	-0.50	(-1.63, 0.64)	0.3911	-0.63	(-1.73, 0.46)	0.2555
Post-T2D MDD duration (years)	0.53	( 0.19, 0.87)	0.0020	0.51	( 0.17, 0.86)	0.0038

The unadjusted model contains the MDD exposure variables and T2D disease durations splines as fixed effects. The adjusted model includes the following additional fixed effects: sex, UKB assessment centre, age at T2D diagnosis, BMI at T2D diagnosis, SBP and DBP at T2D diagnosis, HbA1c at T2D diagnosis, self-reported ethnicity, qualifications at UKB initial assessment, number of observations prior to T2D (BMI, BP and HbA1c), ever smoke, never consumed alcohol, T2D medications and interactions between T2D diagnosis and HbA1c at T2D, T2D medications and BMI. Presented *P*-values are from t-tests. Model parameters and *P*-values from the adjusted model were pooled from 50 MI datasets. Additional file 3: Tables S3 and S4 present full model results for the unadjusted and pooled adjusted model respectively

For post-T2D MDD individuals, the unadjusted model included a small, non-significant decrease in HbA1c of -0.50 mmol/mol (95% CI: [-1.63, 0.64]) upon MDD diagnosis (Table 3). This initial MDD diagnosis effect is retained in the model due to the significant semi-continuous post-T2D MDD duration variable, capturing time since post-T2D MDD diagnosis (Table 3). For two post-T2D MDD individuals where MDD is diagnosed one year apart, HbA1c is expected to be 0.53 mmol/mol higher (95% CI: [0.19, 0.87]) in the individual diagnosed with MDD one year earlier (Table 3).

**Fig. 4:**
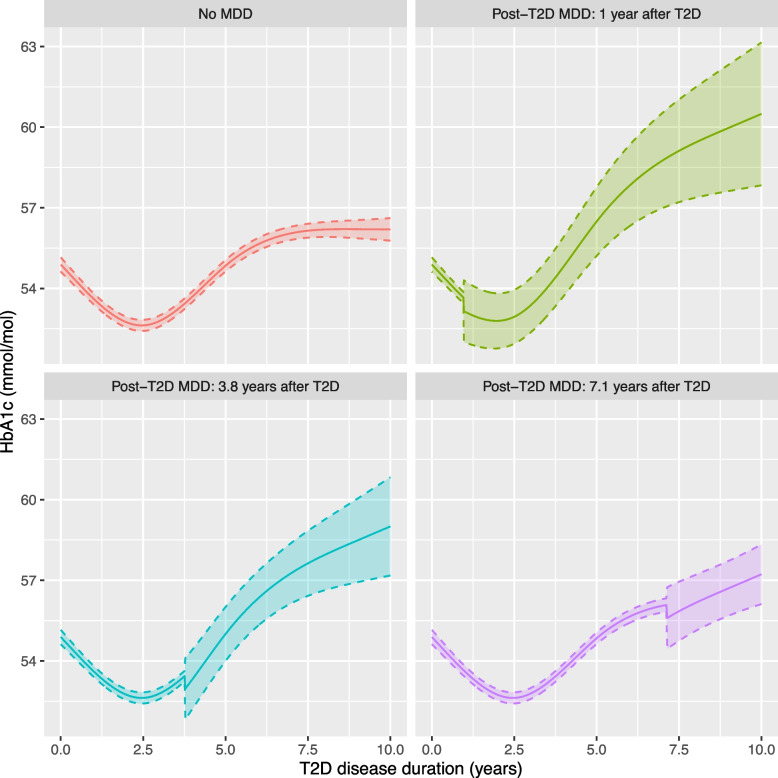
Predicted HbA1c (mmol/mol) with 95% confidence intervals from the unadjusted model, for four example individuals: no MDD and three post-T2D MDD individuals diagnosed with MDD 1 (10th percentile), 3.8 (median) and 7.1 (90th percentile) years after T2D

Figure 4 presents predicted HbA1c over time for an individual without MDD and three example individuals diagnosed with MDD 1 year, 3.8 years and 7.1 years after T2D (10th, 50th and 90th percentile respectively). The smaller sample size for the post-T2D MDD group is reflected in wider 95% CI after MDD diagnosis. An initial decrease in HbA1c at MDD diagnosis is followed by a steeper increase in HbA1c, so that a post-MDD individual will have a higher HbA1c three years after MDD diagnosis compared to an individual with no MDD diagnosis (i.e. the 95% CI for the difference between predicted HbA1c for a post-T2D MDD individual and an individual without MDD is expected to exclude 0 after three years). Individuals diagnosed with MDD earlier in their follow-up are expected to have higher HbA1c levels after a 10-year T2D disease duration compared to those with later onset MDD or those without MDD diagnoses (Additional file 3: Table S2b). For example, at the end of the 10-year follow-up, an individual diagnosed with MDD one year after T2D is predicted to have a HbA1c of 60.49 (95% CI: [57.83, 63.15]) compared to 56.19 (95% CI: [55.78, 56.61]) for those without MDD.

#### Adjusted model

After adjustment for all covariates, the absolute effect sizes for all MDD exposure variables were reduced (Table 3). In particular, the pre-T2D MDD duration effect size is no longer significant (*p* = 0.1637) suggesting that, given the included covariates, a history of MDD prior to T2D has no effect on HbA1c trends over T2D disease duration.

For the post-T2D MDD exposures, the pooled results from the adjusted model are similar to those from the unadjusted model. The adjusted model suggests a small, non-significant decrease in HbA1c after a diagnosis with MDD during follow-up (*p* = 0.2555). The adjusted effect size for post-T2D MDD duration is 0.51 (95% CI: [0.17, 0.86]), showing that earlier MDD diagnosis during follow-up is still associated with higher HbA1c. Full model summaries can be found in Additional file 3: Tables S3 and S4 for the unadjusted and adjusted model respectively.

#### Within-patient HbA1c variability

Non-convergence in the MI datasets was common for models allowing residual variation to differ by MDD_index (24%) and MDD_change (48%). Pooled results from converged models showed that within-patient variability in HbA1c was approximately the same when comparing pre-T2D MDD individuals (using MDD_index) to all others, and post-T2D MDD (using MDD_change) to all others (Table 4). However, when looking retrospectively for post-T2D MDD individuals (i.e. looking across all follow-up rather than only after MDD diagnosis), we found that within-patient HbA1c variation was 1.16 times higher (95% CI: [1.13. 1.19]) for the post-T2D MDD group compared to no MDD and pre-T2D MDD. This suggests that differences in HbA1c for those who develop MDD after T2D, compared to those with no or pre-existing MDD, start prior to MDD diagnosis.

**Table 4:** Pooled hypothesis testing for within-subject variability in HbA1c differing by MDD diagnosis. The presented parameter estimate for each MDD diagnosis variable represents the ratio of the residual variance estimate for the MDD level relative to the reference

MDD diagnosis variable	Reference group(s)	% of MI datasets used	Estimate (95% CI)	Median LRT *P*-value
Pre-T2D MDD (MDD_index)	No MDD, post-T2D MDD	76%	0.98 (0.96, 1.00)	0.0126
Post-T2D MDD (MDD_change)	No MDD, pre-T2D MDD, no post-T2D individuals before MDD diagnosis	52%	0.97 (0.95, 1.00)	0.0873
Post-T2D MDD (retrospective)	No MDD, pre-T2D MDD	100%	1.16 (1.13, 1.19)	1.095E-40

Residual variance (within-subject variation,${\sigma }_{R}^{2}$):${\widehat{\sigma }}_{R}$= 0.54 (95% CI: [0.54, 0.55]). Reference category for each model will have residual variance$={\sigma }_{R}^{2}$. Residual variance form was specified using the varIdent function in the models ‘weights’ input. See Additional file 1: Methods S8 and nlme package help documentation for details. Convergence issues were common when allowing${\sigma }_{R}^{2}$to differ by MDD diagnosis variable- % of MI datasets used column shows the percentage of the 50 MI datasets with no convergence issues

*LRT  *likelihood ratio test

## Discussion

This study tested the hypothesis that people with both T2D and MDD have poorer diabetic control over the course of T2D, as assessed by routine primary care monitoring of HbA1c, focusing on the role of timing and history of MDD diagnosis relative to T2D. We examined whether UKB participants with T2D and MDD have higher and more variable HbA1c levels over time compared to those with T2D alone. To do so, we utilised exposure variables that differentiated between individuals with a history of MDD before their T2D diagnosis (pre-T2D MDD) and those who received their MDD diagnosis after their T2D diagnosis (post-T2D MDD), while also incorporating time since MDD diagnosis.

For individuals with pre-T2D MDD, longitudinal modelling across 10 years of follow-up found that the time between MDD and T2D diagnoses was informative about HbA1c levels across T2D disease duration. Specifically, individuals diagnosed with MDD decades prior to T2D had lower HbA1c over time compared to individuals without MDD and those diagnosed closer to their T2D diagnosis date. After adjusting for covariates, the pre-T2D MDD variables became non-significant, which may be partly ascribed to the correlation between the time gap from pre-T2D MDD diagnosis to T2D and the age at T2D diagnosis (Spearman’s $\rho =0.23$). Here, a longer gap between MDD and T2D diagnoses correlates with a later age at T2D diagnosis (Additional file 3: Table S5). However, the non-significance of pre-T2D MDD exposures after covariate adjustment may also be partly attributed to mediation rather than confounding. For example, increased numbers of pre-T2D measurements for blood pressure, BMI and HbA1c (a proxy for history of healthcare utilisation) were associated with lower HbA1c at T2D diagnosis (Additional file 3: Table S6). Given that an earlier diagnosis with MDD relative to T2D leads to more pre-T2D measurements (Additional file 3: Table S5), it is plausible that mediation is partially responsible for the loss of significance for pre-T2D MDD duration, through increased contact with healthcare professionals. Further work on the impact of pre-T2D MDD is therefore warranted, with extension to additional features of MDD beyond timing of initial diagnosis (e.g. most recent episode, antidepressant prescriptions and number of episodes).

For individuals diagnosed with MDD after T2D, both the unadjusted and adjusted models found that time since MDD diagnosis was important in shaping the trajectory of HbA1c. The earlier the diagnosis of MDD during follow-up, the greater the expected difference in HbA1c between post-T2D MDD individuals and those without MDD at the end of follow-up. Post-T2D individuals also demonstrated some differences in within-patient HbA1c variation. Results suggest that, given the model considered, after diagnosis with MDD during follow-up, within-patient variation does not differ from no MDD and pre-T2D MDD. However, if we look retrospectively across all follow-up and compare post-T2D MDD individuals to all others, we observe that within-patient HbA1c variation is 1.16 times higher for the post-T2D MDD individuals. This greater variability in HbA1c, driven by observations before MDD diagnosis, merits further investigation to ensure appropriate public health and clinical advice is available. Post-T2D MDD individuals may indeed have higher HbA1c variability before MDD diagnosis. This is important because increased variability is associated with increased likelihood of adverse outcomes, including microvascular disease [36]. However, this result may also arise from mean HbA1c trends for post-T2D MDD individuals deviating from those with no or pre-existing MDD before their MDD diagnosis. Given that MDD diagnosis requires the presence of symptoms for two or more weeks, it is plausible that the impact of MDD symptoms on HbA1c begin before formal diagnosis. Additionally, diagnostic delays, as demonstrated in a study of primary care records in Spain (mean delay of 9.89 weeks) [37] may be present. Therefore, exploring the feasibility of using HbA1c trends for MDD prediction in individuals with T2D could be an interesting avenue of research.

Previous work on mean HbA1c has provided inconclusive evidence for the impact of MDD [9]. Nevertheless, in line with our results, two larger studies did find associations between mean HbA1c over time and depression. A meta-analysis found a modest association between depressive symptoms and HbA1c (3-year mean follow-up) [9], and a prospective study in veterans with T2D found that HbA1c was slightly higher for individuals with depression (5-year follow-up) [38].

A previous study [10] showed differences in T2D clinical characteristics by timing of MDD and T2D diagnoses, with a higher rate of diabetic complications in the post-T2D MDD group, but not the pre-TDD MDD group, compared to the no MDD group. Differences in glycaemic control between these MDD subgroups is a possible explanation for the observed clinical differences. The observed increase in HbA1c levels with time since MDD diagnosis for post-T2D MDD individuals, along with the retrospectively identified higher variability across follow-up, supports this hypothesis, demonstrating the potential importance of the relative timing of MDD onset.

Our analysis revealed a linear effect for time since post-T2D MDD diagnosis, meaning the impact of post-T2D MDD increases with years since MDD diagnosis. Due to the smaller sample size of the post-T2D MDD group (*n* = 226), this study may have been underpowered to detect non-linear trends for this post-T2D MDD duration variable (Additional file 1: Methods S10). Future work using larger samples and different methods of capturing the time between T2D and MDD diagnoses (e.g. joint models for HbA1c over time and time-to-MDD diagnosis) may identify such trends and determine if patients diagnosed with MDD after T2D require closer monitoring, or whether a specific time window is crucial for glycaemic control.

MDD episodes after T2D diagnosis are hypothesised to have a greater effect on glycaemic control, which we have confirmed by showing greater mean and within-subject variability in HbA1c levels for post-T2D MDD individuals. These individuals visited their GP after their T2D diagnosis and had a MDD code recorded. Multiple pathways, particularly behavioural, could explain this association. Several studies have shown that patients with both MDD and T2D have worse T2D self-management, are less able to keep the medical appointments, are less physically active and less able to adhere to dietary requirements, possibly leading to hyperglycaemia [39–41]. Our study highlights the importance of diagnosis timing, with possible need for targeted interventions based on clinical history.

This study has several limitations. Firstly, primary care data are collected when patients visit their GP, and between-patient differences in this visiting process can bias results [42, 43]. Individuals with T2D should see their GP every 3–6 months [11] and differences may affect results. For example, less healthy individuals may interact with GPs more frequently and contribute more observations. The models used attempted to reduce this potential bias, as the CAR1 error structure ensured that observations measured closely in time provided less information than those taken far apart. However, T2D cases with MDD may miss more scheduled appointments, and our analysis does not account for this.

Secondly, as a retrospective observational study, confounding may bias results. For example, antidepressant medication can lead to weight gain [44], which can negatively affect HbA1c levels [45]. While we adjusted for confounders such as BMI and blood pressure, these covariates were time invariant, and the timing of measurement differed for each participant (Additional file 1: Methods S6). Future studies could consider time-varying confounding and include additional risk factors (e.g. antidepressant medications, cardiovascular conditions).

Thirdly, HbA1c at T2D diagnosis had a high level of missingness, which disproportionately affected individuals in the post-T2D MDD group and highlights the challenges of working with real-world data. While we utilised MI, this approach has limitations and depends on the model selected and data availability. However, results for the post-T2D MDD exposures were similar in the unadjusted model (no MI required) and the adjusted model (MI used).

The validity of the age at MDD onset, determined using the date of the first MDD diagnostic code, presents a further limitation. For older individuals the validity of their MDD onset is unknown, given primary care records are only available after 1990, while mean age at diagnosis of MDD is around 30 years [46]. Replication in the Clinical Practice Research Datalink, which has GP records from 1987 and no age limits, would therefore be useful [47].

## Conclusions

This study, utilising UKB primary care records, highlights the importance of considering the temporal relationships between T2D and MDD in the context of glycaemic control. Findings reveal a non-linear trend in HbA1c levels over time and demonstrated that within a 10-year window, those who develop MDD after T2D diagnosis had increased HbA1c levels and, prior to MDD diagnosis, greater variability, supporting the finding that individuals with both conditions have poorer health outcomes. Regular routine screening for depression, integrated mental health support and closer monitoring may reduce the adverse consequences associated with both T2D and MDD.

### Supplementary Information

Additional file 1: Supplementary methods. Methods S1- Participant-level QC for analysis with genetic data. Methods S2- Diabetic medicine extraction and variable creation. Methods S3- T2D diagnosis exclusion criteria. Methods S4- Valid HbA1c. Methods S5- MDD diagnosis. Methods S6- BMI, SBP and DBP at T2D diagnosis. Methods S7- T2D polygenic score. Methods S8- Residual within-subject variability analysis. Methods S9- Multiple imputation. Methods S10- Non-linear time-trend for post-T2D MDD individuals.

Additional file 2: Supplementary Tables for the Methods section of the main paper. Table S1- GP codes for diabetes. Table S2- HES codes for diabetes (ICD9 and 10 codes). Table S3- Glucose lowering medication codes. Table S4- HbA1c read 2 and read 3 codes. Table S5- Sensitivity analysis excluding covariates not measured approximately at T2D diagnosis. Table S6- Details on all variables extracted for this analysis. Table S7- Description of which variables are used in the fixed effects model for all models run in this study.

Additional file 3: Supplementary Tables for the Results section of the main paper. Table S1- Validation of T2D diagnosis: Association between T2D case-control status and T2D polygenic scores. Table S2- Predicted HbA1c values (mmol/mol) over time (years), with 95% CIs, from the unadjusted model for a selection of hypothetical example individuals. Table S3- Unadjusted model summary. Table S4- Adjusted model summary (pooled from 50 MI datasets). Table S5- Exploring the relationship between pre-T2D MDD duration, up to T2D diagnosis, and covariates. Table S6- Characterising those missing HbA1c at T2D diagnosis.

Additional file 4: Supplementary Figures S1-S8. All figures present a density plot for a continuous variable stratified by MDD subgroup. Figure S1- TDI (at UKB initial assessment). Figure S2- Age (years) at T2D diagnosis. Figure S3- Follow-up time (years). Figure S4- Total number of observations prior to T2D diagnosis. Figure S5- BMI at T2D diagnosis. Figure S6- SBP at T2D diagnosis. Figure S7- DBP at T2D diagnosis. Figure S8- HbA1c (mmol/mol) at T2D diagnosis.

## Data Availability

The data used in this study are available for approved researchers from the UK Biobank. Permissions are required to access UK Biobank data. Researchers can register and apply at http://www.ukbiobank.ac.uk/register-apply. R code used for data management and analysis are available here: https://github.com/alexgillett/MDD_T2D_HbA1c_LMM.

## Abbreviations

HbA1c: Glycated haemaglobin
T2D: Type 2 diabetes
MDD: Major depressive disorder
UKB: UK Biobank
GP: General practioner
PGS: Polygenic score
BMI: Body mass index
SBP: Systolic blood pressure
DBP: Diastolic blood pressure
MEM: Mixed effects model
CAR1: Continuous-time autoregressive 1
LRT: Likelihood ratio test
MI: Multiple imputation
CI: Confidence interval
IQR: Interquartile range
HES: Hospital episode statistics

## References

[1] Holt RIG, de Groot M, Golden SH (2014). Diabetes and Depression. Curr DiabRep.

[2] Knol MJ, Twisk JWR, Beekman ATF, Heine RJ, Snoek FJ, Pouwer F (2006). Depression as a risk factor for the onset of type 2 diabetes mellitus. A meta-analysis Diabetologia.

[3] Mezuk B, Eaton WW, Albrecht S, Golden SH (2008). Depression and Type 2 Diabetes Over the Lifespan. Diabetes Care.

[4] Nouwen A, Adriaanse MC, van Dam K, Iversen MM, Viechtbauer W, Peyrot M (2019). Longitudinal associations between depression and diabetes complications: a systematic review and meta-analysis. Diabet Med.

[5] Wu C, Hsu L, Wang S. Association of depression and diabetes complications and mortality: a population-based cohort study. epidemiology and psychiatric sciences 2020;29. 10.1017/S2045796020000049. 10.1017/S2045796020000049 PMC7214709 31992379

[6] Martín-Timón I, Sevillano-Collantes C, Segura-Galindo A, Del Cañizo-Gómez F (2014). Type 2 diabetes and cardiovascular disease: Have all risk factors the same strength?. World J Diabetes.

[7] Gorst C, Kwok CS, Aslam S, Buchan I, Kontopantelis E, Myint PK (2015). Long-term Glycemic Variability and Risk of Adverse Outcomes: A Systematic Review and Meta-analysis. Diabetes Care.

[8] Sherwani S, Khan H, Ekhzaimy A, Masood A, Sakharkar M (2016). Significance of HbA1c Test in Diagnosis and Prognosis of Diabetic Patients. Biomarker Insight.

[9] Beran M, Muzambi R, Geraets A, Albertorio-Diaz JR, Adriaanse MC, Iversen MM (2022). The bidirectional longitudinal association between depressive symptoms and HbA1c : A systematic review and meta-analysis. Diabet Med.

[10] Bruce DG, Davis WA, Cetrullo V, Starkstein SE, Davis TME (2013). Clinical Impact of the Temporal Relationship between Depression and Type 2 Diabetes: The Fremantle Diabetes Study Phase II. PLoS ONE.

[11] McGuire H, Longson D, Adler A, Farmer A, Lewin I (2016). Management of type 2 diabetes in adults: summary of updated NICE guidance. BMJ.

[12] Sudlow C, Gallacher J, Allen N, Beral V, Burton P, Danesh J (2015). UK Biobank: An Open Access Resource for Identifying the Causes of a Wide Range of Complex Diseases of Middle and Old Age. PLoS Med.

[13] The UK Biobank. Primary Care Linked Data 2024. https://biobank.ndph.ox.ac.uk/showcase/showcase/docs/primary_care_data.pdf.

[14] Hagenaars SP, Coleman JRI, Choi SW, Gaspar H, Adams MJ, Howard DM (2020). Genetic comorbidity between major depression and cardio-metabolic traits, stratified by age at onset of major depression. Am J Med Genet B Neuropsychiatr Genet.

[15] Bycroft C, Freeman C, Petkova D, Band G, Elliott LT, Sharp K (2018). The UK Biobank resource with deep phenotyping and genomic data. Nature.

[16] Farmer RE, Beard I, Raza SI, Gollop ND, Patel N, Tebboth A (2021). Prescribing in Type 2 Diabetes Patients With and Without Cardiovascular Disease History: A Descriptive Analysis in the UK CPRD. Clin Ther.

[17] Holm N-CR, Belstrøm D, Østergaard JA, Schou S, Holmstrup P, Grauballe MB. Identification of individuals with undiagnosed diabetes and pre-diabetes in a Danish cohort attending dental treatment. J Periodontol 2016;87:395–402. 10.1902/jop.2016.150266. 10.1902/jop.2016.150266 26745612

[18] Fabbri C, Hagenaars SP, John C, Williams AT, Shrine N, Moles L (2021). Genetic and clinical characteristics of treatment-resistant depression using primary care records in two UK cohorts. Mol Psychiatry.

[19] NGSP. IFCC Standardization of HbA1c n.d. http://www.ngsp.org/ifccngsp.asp.

[20] Young KG, McDonald TJ, Shields BM (2022). Glycated haemoglobin measurements from UK Biobank are different to those in linked primary care records: implications for combining biochemistry data from research studies and routine clinical care. Int J Epidemiol.

[21] Ko S, German CA, Jensen A, Sinsheimer JS, Zhou H, Zhou JJ (2022). GWAS of longitudinal trajectories at biobank scale. AJHG.

[22] Bhaskaran K, Forbes HJ, Douglas I, Leon DA, Smeeth L. Representativeness and optimal use of body mass index (BMI) in the UK Clinical Practice Research Datalink (CPRD). BMJ Open 2013;3. 10.1136/bmjopen-2013-003389. 10.1136/bmjopen-2013-003389 PMC3773634 24038008

[23] Hanscombe K. ukbkings: KCL interface to UKB Project Data on Rosalind/CREATE HPC. 2022. Available online: https://kenhanscombe.github.io/ukbkings/.

[24] Scott RA, Scott LJ, Mägi R, Marullo L, Gaulton KJ, Kaakinen M (2017). An Expanded Genome-Wide Association Study of Type 2 Diabetes in Europeans. Diabetes.

[25] Choi SW, O’Reilly PF. PRSice-2: Polygenic Risk Score software for biobank-scale data. GigaScience 2019;8:giz082. 10.1093/gigascience/giz082. 10.1093/gigascience/giz082 PMC6629542 31307061

[26] Choi SW, Mak TS-H, O’Reilly PF. Tutorial: a guide to performing polygenic risk score analyses. Nature Protocols 2020;15:2759–72. 10.1038/s41596-020-0353-1. 10.1038/s41596-020-0353-1 PMC7612115 32709988

[27] Pinheiro J, Bates D (2000). Mixed-Effects Models in S and S-PLUS.

[28] Pinheiro J, Bates D, R Core Team. nlme: Linear and nonlinear mixed effects models. 2022. R package version 3.1-159. https://CRAN.R-project.org/package=nlme.

[29] Harrell F. Regression Modeling Strategies: With applications to linear models, logistic regression, and survival analysis. New York: Springer-Verlag; 2001. 10.1007/978-1-4757-3462-1.

[30] Gauthier J, Wu QV, Gooley TA (2020). Cubic splines to model relationships between continuous variables and outcomes: a guide for clinicians. Bone Marrow Transplant.

[31] Honaker J, King G, Blackwell M (2011). Amelia II: A Program for Missing Data. J Stat Soft.

[32] Li K-H, Meng X-L, Raghunathan TE, Rubin DB. Significance levels from repeated p-values with multiply-imputed data. Stat Sin. 1991;1:65–92. http://www.jstor.org/stable/24303994.

[33] Marshall A, Altman DG, Holder RL, Royston P (2009). Combining estimates of interest in prognostic modelling studies after multiple imputation: current practice and guidelines. BMC Med Res Methodol.

[34] Shepherd BE, Rebeiro PF, the Caribbean C and SA network for H epidemiology. Brief report: assessing and interpreting the association between continuous covariates and outcomes in observational studies of HIV using splines. J Acquir Immune Defic Syndr 2017;74. 10.1097/QAI.0000000000001221. 10.1097/QAI.0000000000001221 PMC5303133 27798430

[35] Perperoglou A, Sauerbrei W, Abrahamowicz M, Schmid M (2019). A review of spline function procedures in R. BMC Med Res Methodol.

[36] Škrha J, Šoupal J, Škrha J, Prázný M (2016). Glucose variability, HbA1c and microvascular complications. Rev Endocr Metab Disord.

[37] Huerta-Ramírez R, Bertsch J, Cabello M, Roca M, Haro JM, Ayuso-Mateos JL (2013). Diagnosis delay in first episodes of major depression: A study of primary care patients in Spain. J Affect Disord.

[38] Richardson LK, Egede LE, Mueller M, Echols CL, Gebregziabher M (2008). Longitudinal effects of depression on glycemic control in veterans with Type 2 diabetes. Gen Hosp Psychiatry.

[39] Schmitt A, Reimer A, Hermanns N, Kulzer B, Ehrmann D, Krichbaum M (2017). Depression is linked to hyperglycaemia via suboptimal diabetes self-management: A cross-sectional mediation analysis. J Psychosom Res.

[40] Lustman PJ, Clouse RE (2005). Depression in diabetic patients: The relationship between mood and glycemic control. J Diabetes Complications.

[41] Gonzalez JS, Peyrot M, McCarl LA, Collins EM, Serpa L, Mimiaga MJ (2008). Depression and Diabetes Treatment Nonadherence: A Meta-Analysis. Diabetes Care.

[42] Lokku A, Lim LS, Birken CS, Pullenayegum EM, on behalf of the TARGet Kids! Collaboration. Summarizing the extent of visit irregularity in longitudinal data. BMC Medical Research Methodology 2020;20:135. 10.1186/s12874-020-01023-w. 10.1186/s12874-020-01023-w PMC7260811 32471357

[43] Kalia S, Saarela O, Escobar M, Moineddin R, Greiver M (2023). Estimation of marginal structural models under irregular visits and unmeasured confounder: calibrated inverse probability weights. BMC Med Res Methodol.

[44] Gafoor R, Booth HP, Gulliford MC. Antidepressant utilisation and incidence of weight gain during 10 years’ follow-up: population based cohort study. BMJ 2018;361. 10.1136/bmj.k1951. 10.1136/bmj.k1951 PMC5964332 29793997

[45] Gummesson A, Nyman E, Knutsson M, Karpefors M (2017). Effect of weight reduction on glycated haemoglobin in weight loss trials in patients with type 2 diabetes. Diabetes Obes Metab.

[46] Kessler RC, Berglund P, Demler O, Jin R, Merikangas KR, Walters EE (2005). Lifetime Prevalence and Age-of-Onset Distributions of DSM-IV Disorders in the National Comorbidity Survey Replication. Arch Gen Psychiatry.

[47] Herrett E, Gallagher AM, Bhaskaran K, Forbes H, Mathur R, van Staa T (2015). Data Resource Profile: Clinical Practice Research Datalink (CPRD). Int J Epidemiol.

[48] King’s College London. King’s Computational research, engineering and technology environment (CREATE). 2022. 10.18742/rnvf-m076.

