# Supplementary material for: The impact of major depressive disorder on glycaemic control in type 2 diabetes: a longitudinal cohort study using UK Biobank primary care records
Source: BMC Med. 2024 May 29;22:211. doi: 10.1186/s12916-024-03425-9 (PMC11134616; doi:10.1186/s12916-024-03425-9)

Supplementary Figures

Table of content

	Page
**Plots corresponding to Descriptive Analysis**	
S1: Density plot of TDI by MDD subgroup.	2
S2: Density plot of age (years) at T2D diagnosis by MDD subgroup.	3
S3: Density plots stratified by MDD subgroup for follow-up time (T2D disease duration in years).	4
S4: Density plots stratified by MDD subgroup for number of pre-T2D measurements.	5
S5: Density plot of approximate BMI at T2D diagnosis by MDD subgroup.	6
S6: Density plot of approximate SBP at T2D diagnosis by MDD subgroup.	7
S7: Density plot of approximate DBP at T2D diagnosis by MDD subgroup.	8
S8: Density plot of HbA1c (mmol/mol) at T2D diagnosis by MDD subgroup.	9

Descriptive plots

Supplementary Figure S1: Density plot of Townsend Deprivation Index (TDI) measured at initial UK Biobank assessment, by MDD subgroup.

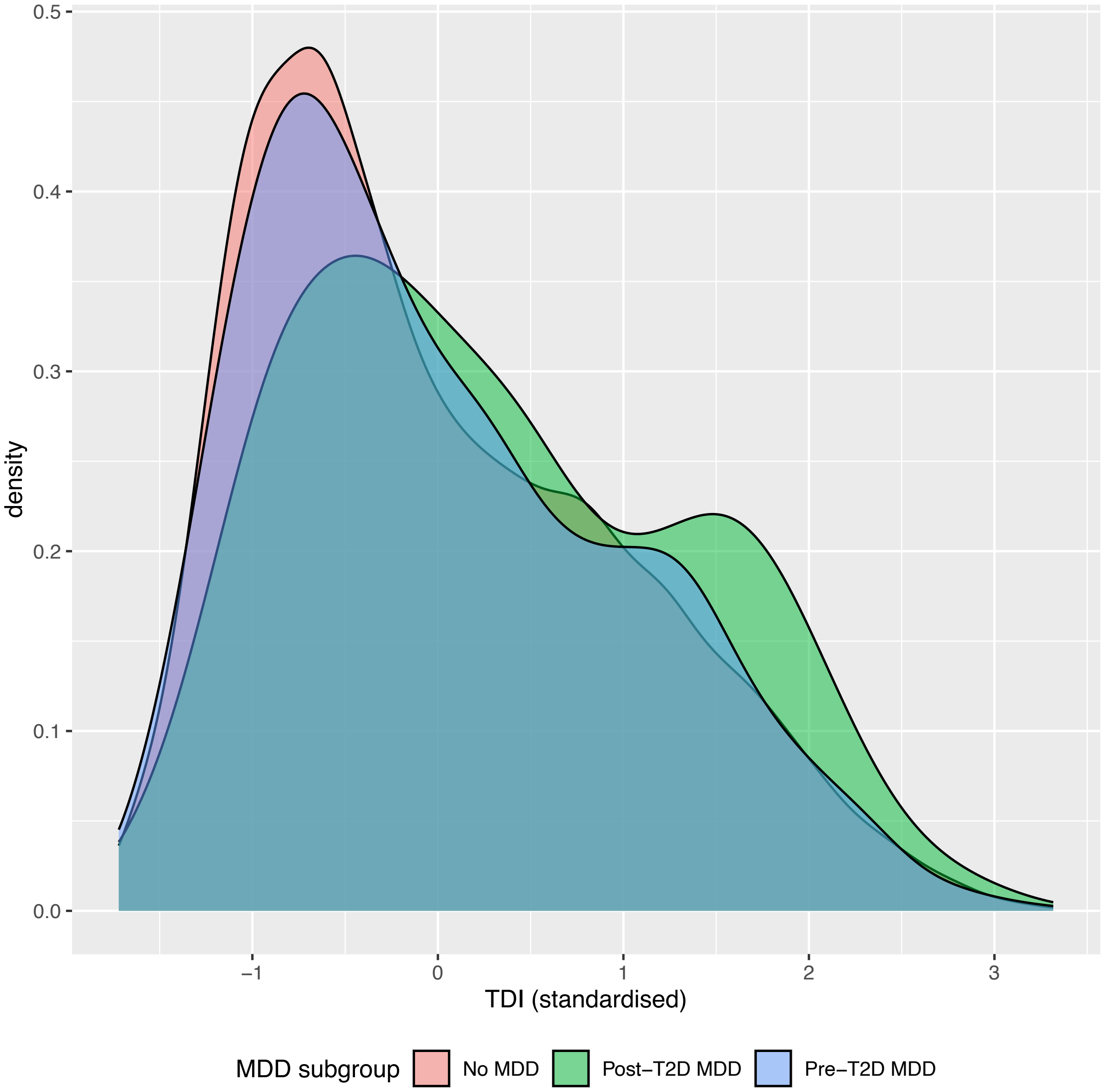

Supplementary Figure S2: Density plot of age (years) at T2D diagnosis by MDD subgroup.

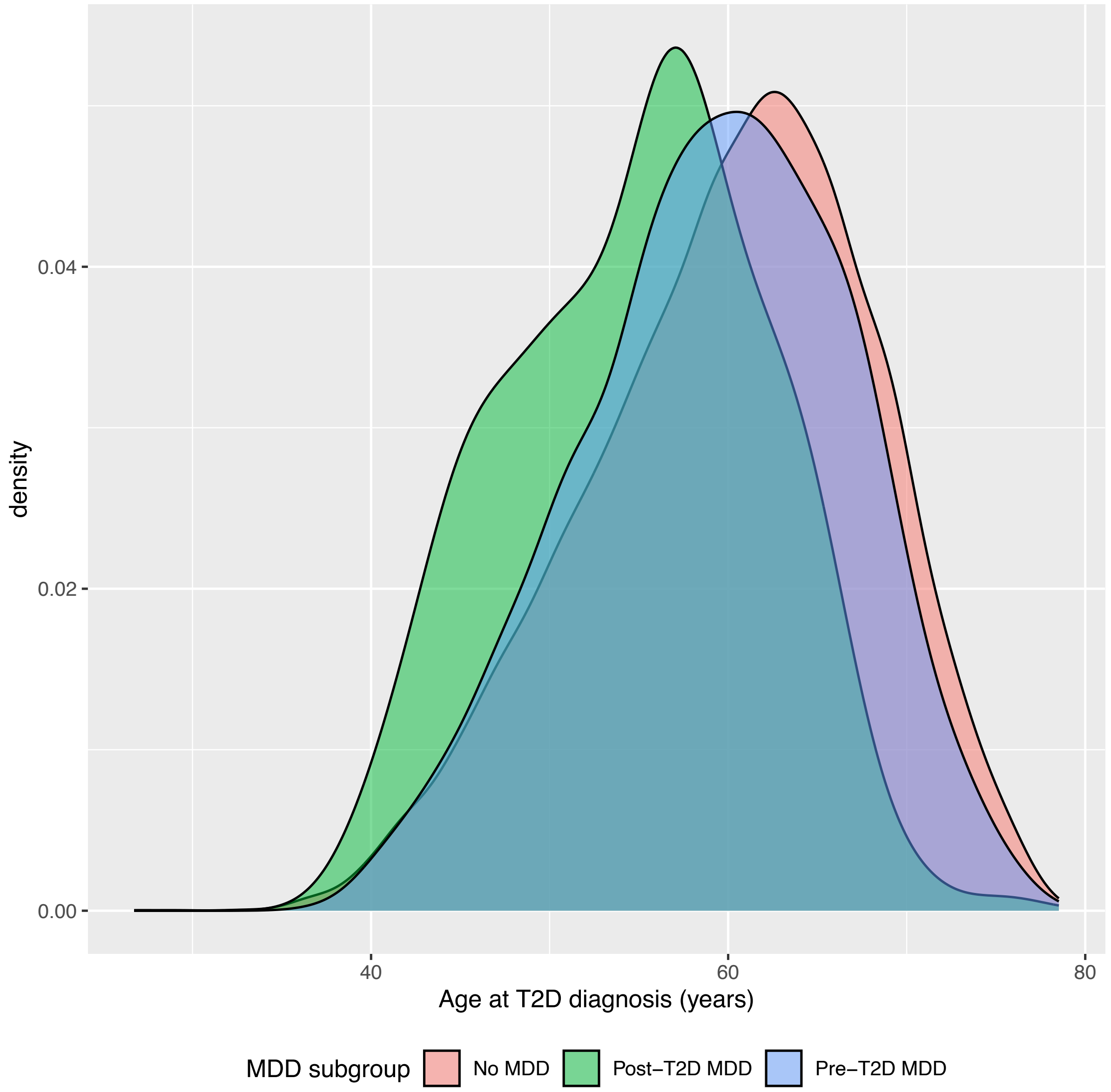

Supplementary Figure S3: Density plots stratified by MDD subgroup for follow-up time (T2D disease duration in years) up to 10 years after T2D diagnosis (end of study).

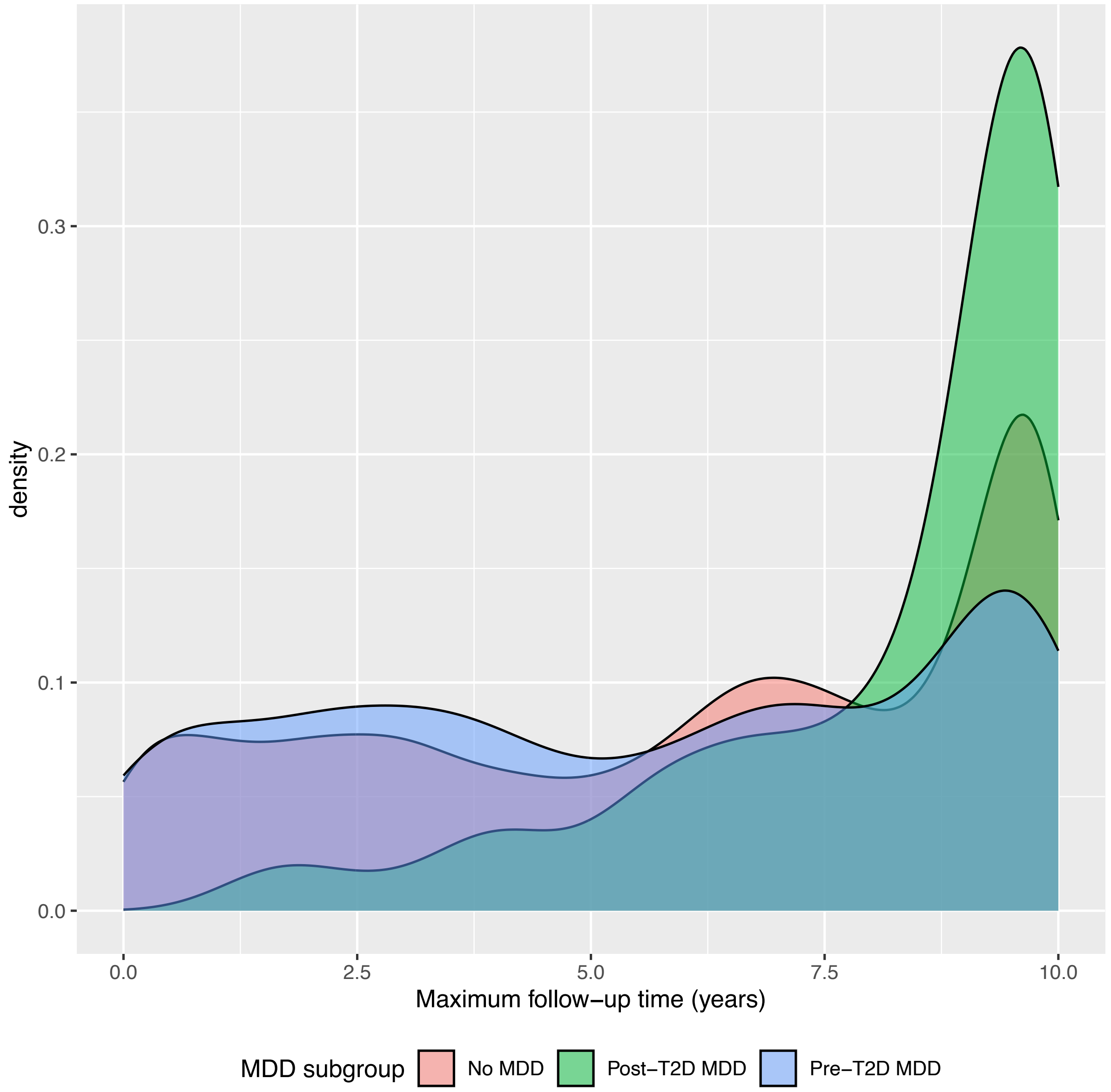

Supplementary Figure S4: Density plots stratified by MDD subgroup for total number of HbA1c, BMI and blood pressure measurements prior to T2D diagnosis.

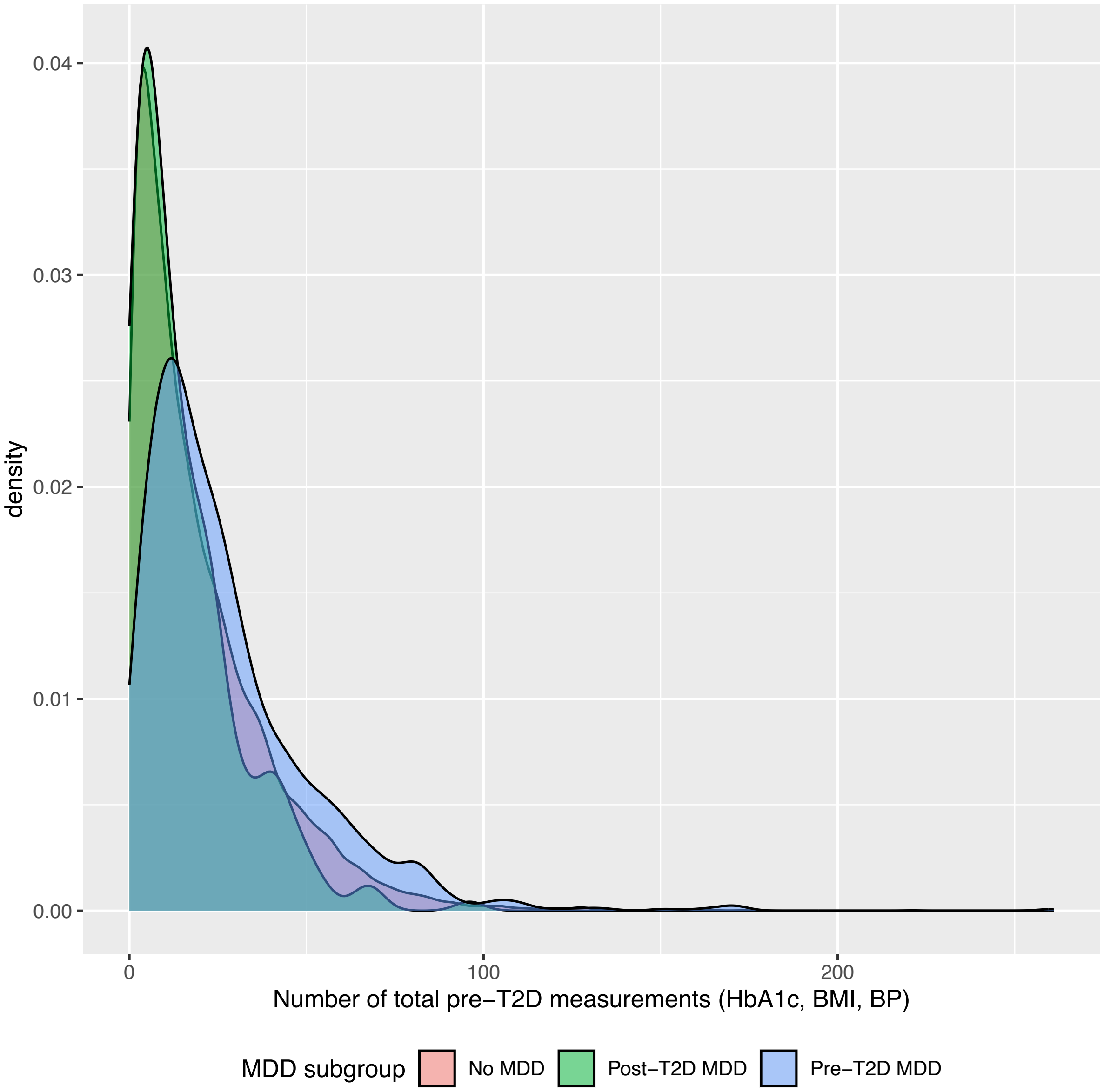

Supplementary Figure S5: Density plot of BMI (approximately) at T2D diagnosis by MDD subgroup.

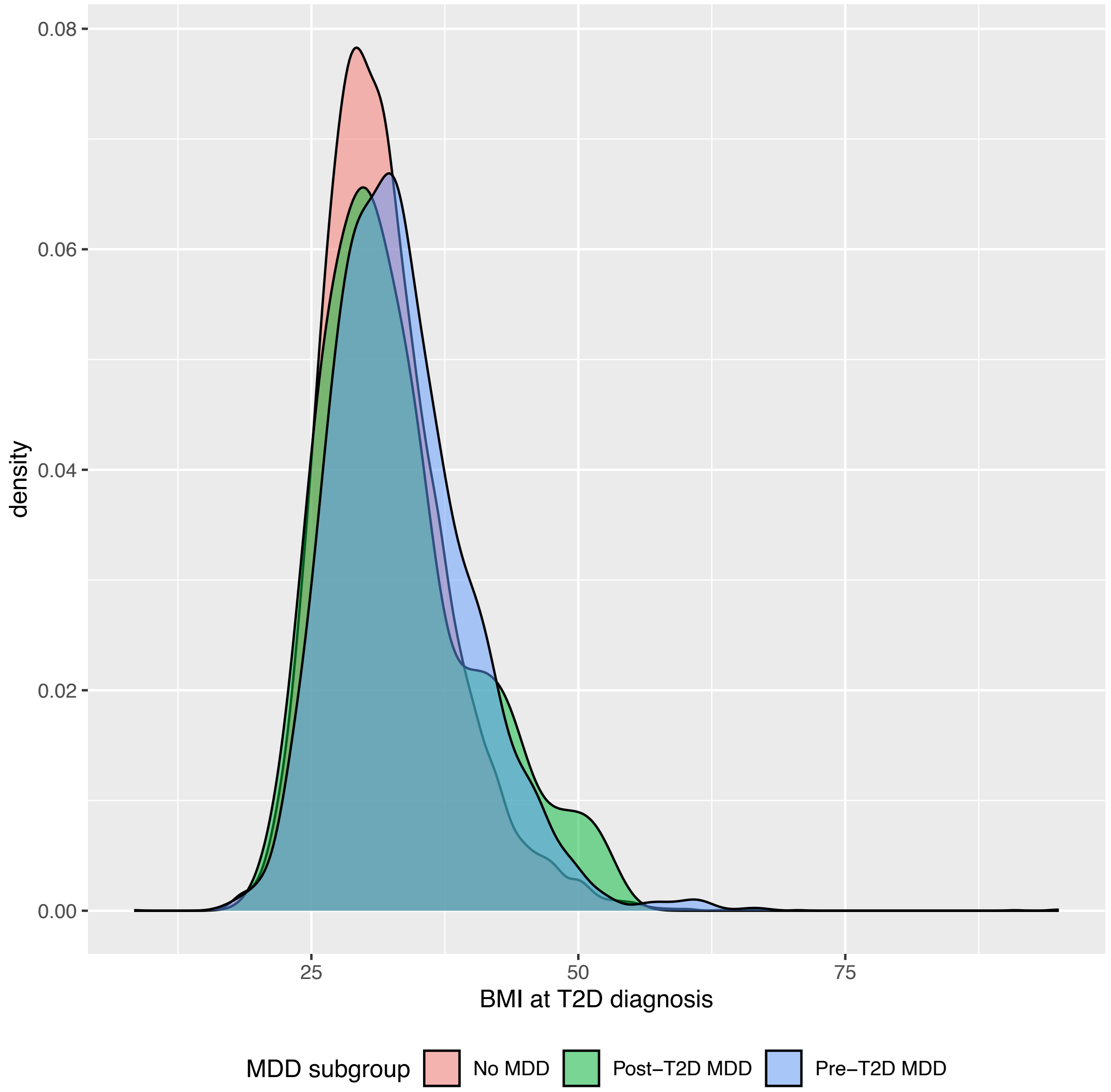

Supplementary Figure S6: Density plot of systolic blood pressure (approximately) at T2D diagnosis by MDD subgroup.

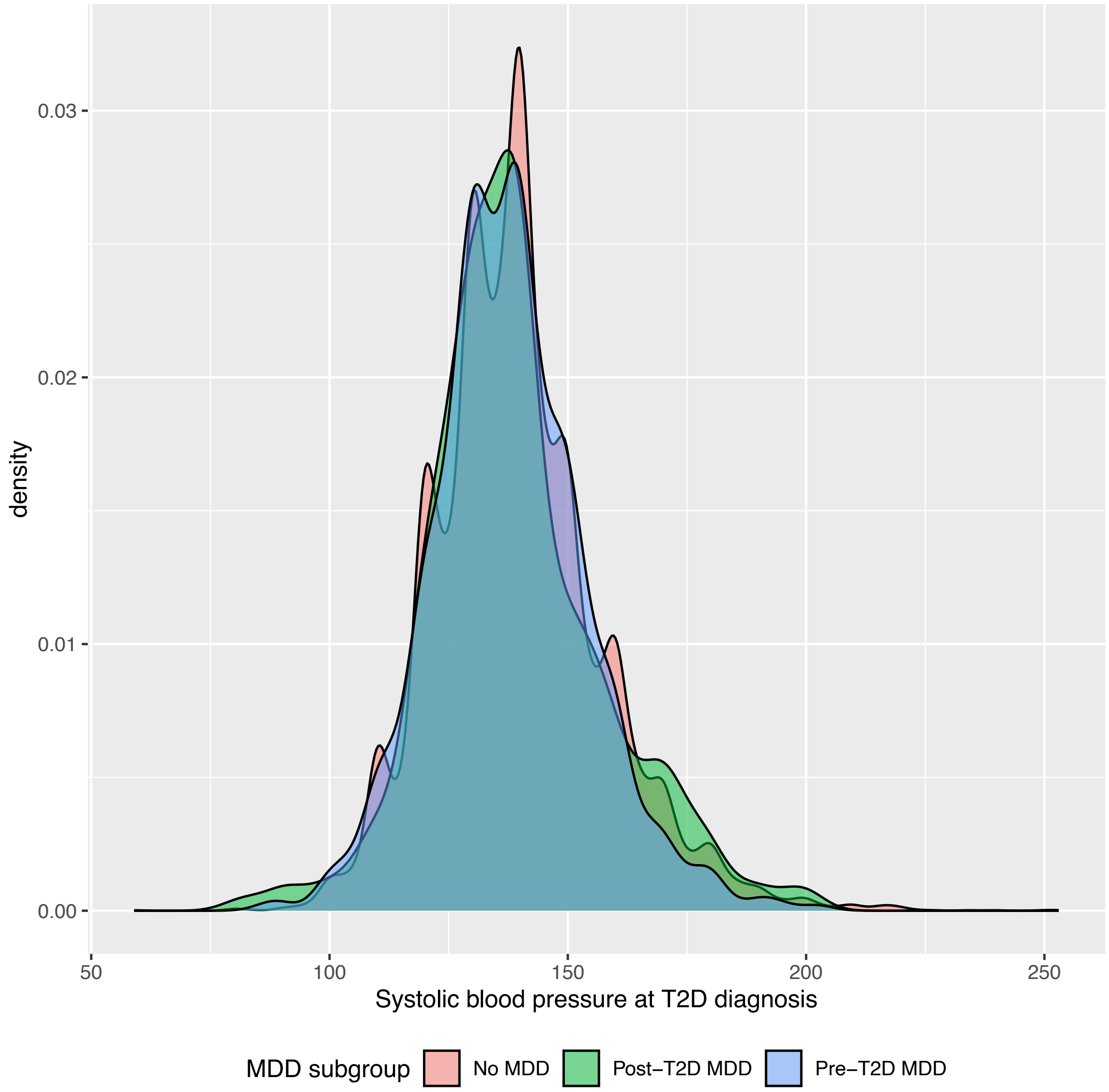

Supplementary Figure S7: Density plot of diastolic blood pressure (approximately) at T2D diagnosis by MDD subgroup.

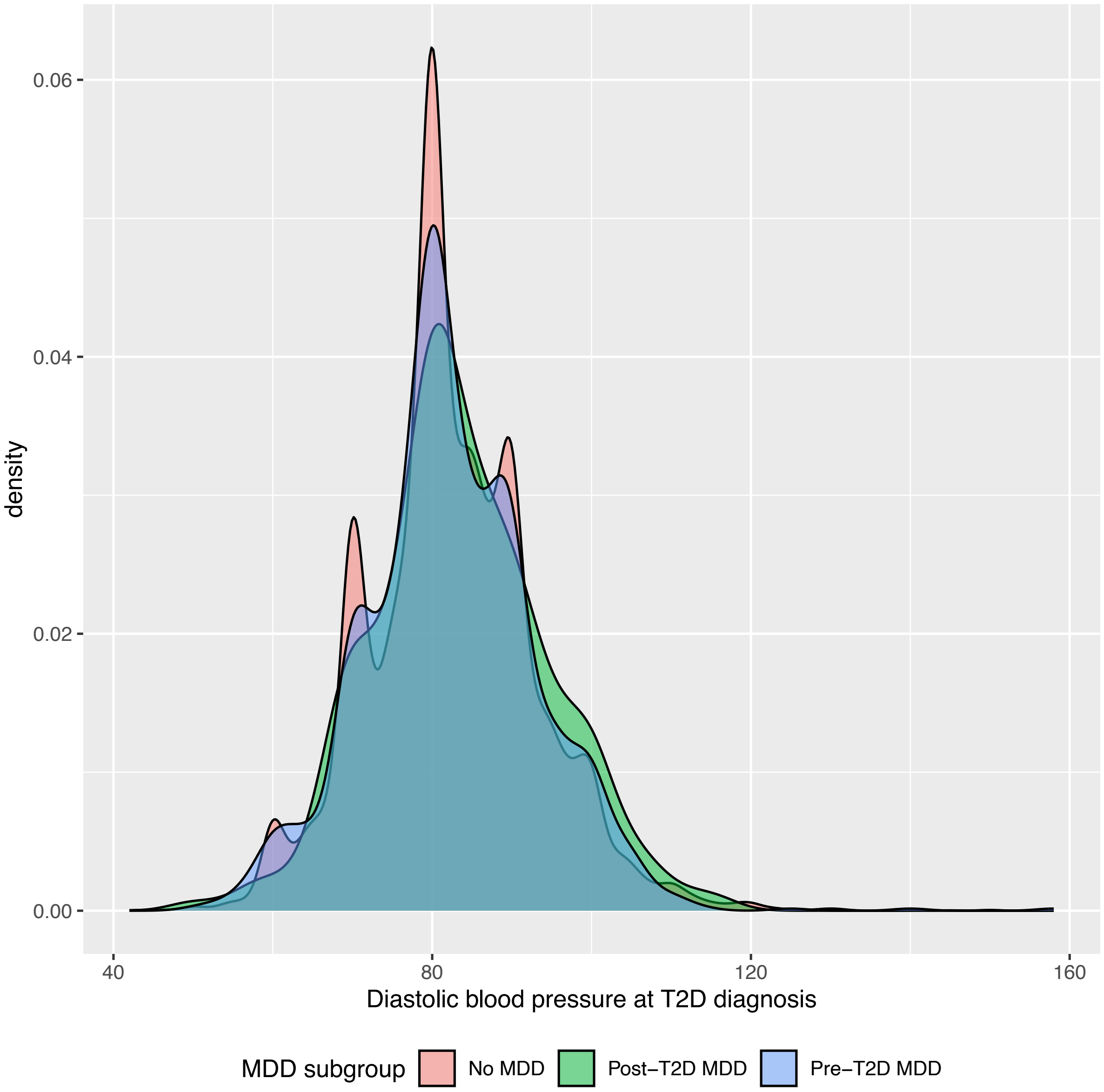

Supplementary Figure S8: Density plot of HbA1c (mmol/mol) at T2D diagnosis by MDD subgroup.

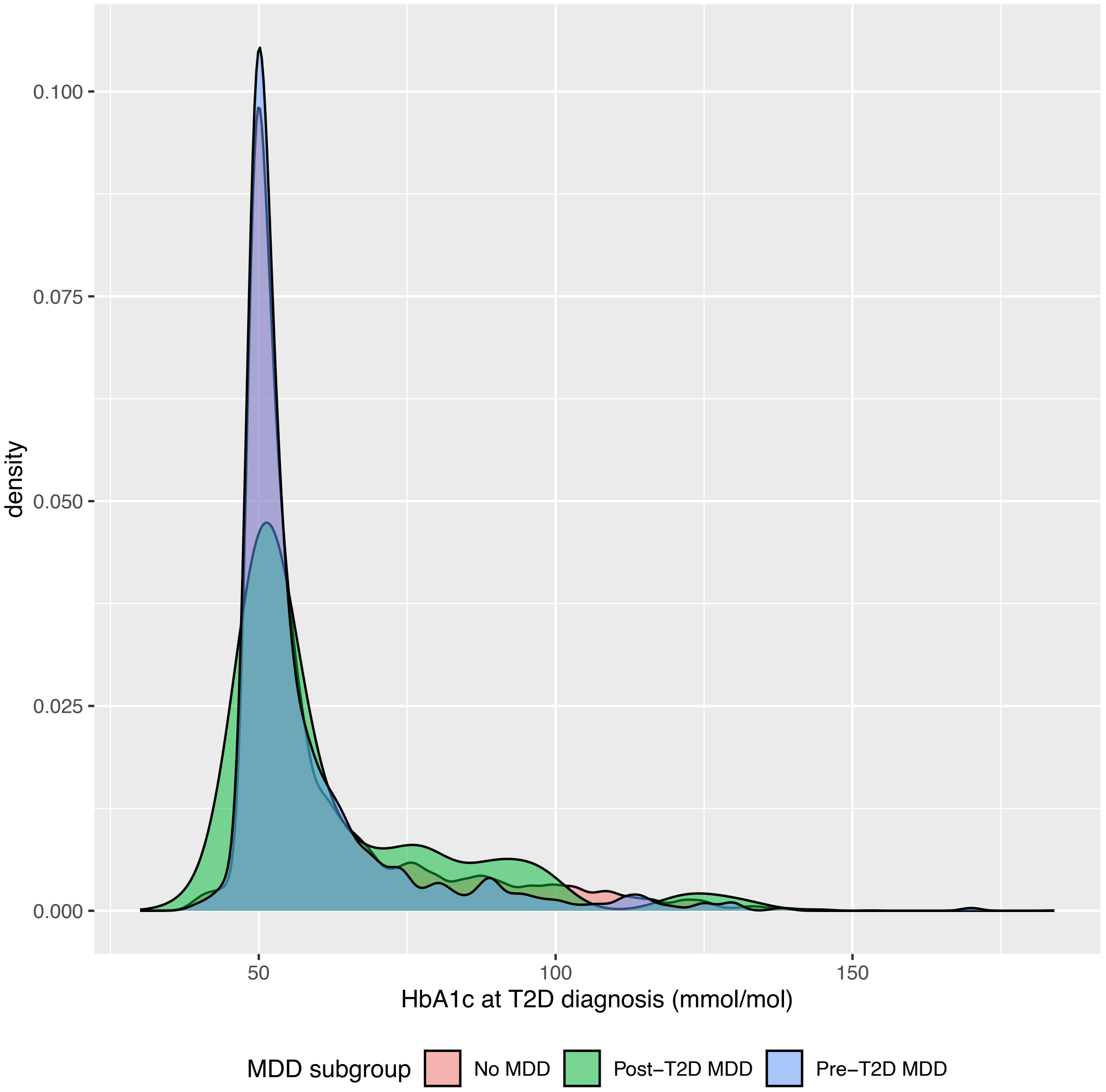

Supplement: Supplementary file 4 — Additional file 4: Supplementary Figures S1-S8. All figures present a density plot for a continuous variable stratified by MDD subgroup. Figure S1- TDI (at UKB initial assessment). Figure S2- Age (years) at T2D diagnosis. Figure S3- Follow-up time (years). Figure S4- Total number of observations prior to T2D diagnosis. Figure S5- BMI at T2D diagnosis. Figure S6- SBP at T2D diagnosis. Figure S7- DBP at T2D diagnosis. Figure S8- HbA1c (mmol/mol) at T2D diagnosis. [file 12916_2024_3425_MOESM4_ESM.pdf]
